# Successful metabolic adaptations leading to the prevention of high fat diet-induced murine cardiac remodeling

**DOI:** 10.1186/s12933-015-0286-0

**Published:** 2015-09-25

**Authors:** Nathan W. Roberts, Magdalis González-Vega, Tirsit K. Berhanu, Aaron Mull, Jesús García, Ahlke Heydemann

**Affiliations:** The University of Illinois at Chicago, COMRB 2035, MC 901, 835 South Wolcott Ave., Chicago, IL 60612-7352 USA; The Center for Cardiovascular Research, Chicago, IL 60612 USA

**Keywords:** High fat diet, Diabetic cardiomyopathy, MRL mouse, pAMPK

## Abstract

**Background:**

Cardiomyopathy is a devastating complication of obesity and type 2 diabetes mellitus (T2DM). It arises even in patients with normoglycemia (glycosylated hemoglobin, A1C ≤7 %). As obesity and T2DM are approaching epidemic levels worldwide, the cardiomyopathy associated with these diseases must be therapeutically addressed. We have recently analyzed the systemic effects of a 12-week high fat diet (HFD) on wild type mice from the C57Bl/6 (B6) strain and the wild type super-healing Murphy Roths Large (MRL) mouse strain. The MRL HFD mice gained significantly more weight than their control diet counterparts, but did not present any of the other usual systemic T2DM phenotypes.

**Methods:**

Cardiac pathology and adaptation to HFD-induced obesity in the MRL mouse strain compared to the HFD C57Bl/6 mice were thoroughly analyzed with echocardiography, histology, qPCR, electron microscopy and immunoblots.

**Results:**

The obese HFD C57Bl/6 mice develop cardiac hypertrophy, cardiomyocyte lipid droplets, and initiate an ineffective metabolic adaptation of an overall increase in electron transport chain complexes. In contrast, the obese HFD MRL hearts do not display hypertrophy nor lipid droplets and their metabolism adapts quite robustly by decreasing pAMPK levels, decreasing proteins in the carbohydrate metabolism pathway and increasing proteins utilized in the β-oxidation pathway. The result of these metabolic shifts is the reduction of toxic lipid deposits and reactive oxygen species in the hearts of the obese HFD fed MRL hearts.

**Conclusions:**

We have identified changes in metabolic signaling in obese HFD fed MRL mice that confer resistance to diabetic cardiomyopathy. The changes include a reduction of cardiac pAMPK, Glut4 and hexokinase2 in the MRL HFD hearts. Overall the MRL hearts down regulate glucose metabolism and favor lipid metabolism. These adaptations are essential to pursue for the identification of novel therapeutic targets to combat obesity related cardiomyopathy.

## Background

Human diabetic cardiomyopathy (DCM) is classified as left ventricular dysfunction without the usual predictors of cardiomyopathy such as hypertension or coronary artery disease [1, 2]. DCM is enigmatic because even diabetic patients with normoglycemia have an increased incidence of DCM [3, 4], and pre-diabetic patients are also prone to DCM [5, 6]. In addition, standard type 2 diabetes mellitus (T2DM) treatments and preclinical experiments often reduce blood glucose and A1C levels but do not prevent DCM and sometimes even increase cardiac pathology [7]. Therefore the etiology of DCM is difficult to identify. Possible etiologic factors combining to cause DCM include obesity, hyperglycemia, oxidative stress, lipotoxicity, inflammation, decreased autophagy and unsuccessful metabolic adaptation [7].

High fat diet (HFD)-fed mice recapitulate the etiology, pathology, and response to treatments similarly to those of T2DM patients. HFD-fed C57Bl/6 (B6) mice gain weight, develop insulin resistance, hyperglycemia, and DCM just like patients do. HFD and obesity in mice lead to cardiac remodeling, which is detected as increased heart mass, increased septal cross-section, increased cardiac fibrosis, and decreased cardiac function [8]. Because MRL mice are globally resistant to HFD pathologies [9], we wanted to determine whether MRL mice develop cardiomyopathy from HFD-induced obesity and possible compensatory protective mechanisms if they did not.

Both T2DM humans and HFD-fed mice respond well to increases in activated AMP-activated protein kinase (pAMPK), which reduces most of the HFD-mediated pathologies [7, 10, 11]. Increasing pAMPK is the target of multiple T2DM therapies, such as metformin, thiazolidines, and exercise. However, the heart does not benefit from metformin treatment and many patients develop DCM despite maintaining normoglycemia [3, 4, 7, 12]. The cardiac pathology of T2DM is therefore clearly separate from the systemic pathology, many patients with normoglycemia still develop DCM, many T2DM patients die of cardiovascular diseases [7]. Therefore, this aspect of T2DM must be further investigated. The obese HFD fed MRL mice, which we now show are resistant to obesity-related cardiac remodeling, will provide additional insights into the prevention of obesity-induced cardiac pathology.

The obese HFD MRL mice remain pathology free despite having gained as much weight as the HFD B6 mice [9]. The HFD MRL mice do not become hyperglycemic, and respond normally to insulin and glucose tolerance tests. The control diet (CD) MRL skeletal muscles contain increased basal AMPK and pAMPK [13]. The HFD MRL skeletal muscles respond and adapt to the HFD by increasing their AMPK and pAMPK levels even more than the basal levels [9]. pAMPK is central to skeletal muscle metabolism at a number of important decision points [14]. During either fasting or routine exercise regimes, when the skeletal muscle’s ATP levels decrease, AMPK is activated by phosphorylation and initiates catabolism to increase ATP levels and inhibits anabolism to conserve ATP. As part of the catabolic processes, increased pAMPK, causes Glut4 translocation to the cellular membrane which will increase glucose internalization [15], causes increased levels of glycolysis [16], which can increase ATP production without requiring oxygen and also causes increases in β-oxidation rates [17]. All of these pAMPK-elicited metabolic changes benefit patients with T2DM.

The 12 weeks HFD B6 control mouse hearts display essential aspects of DCM. These mouse hearts, as a whole, and as individual cardiomyocytes, develop hypertrophy. No molecular attempts to compensate for the HFD diet are displayed by the B6 cohort; therefore the hearts develop lipid deposits and fibrosis. Alternatively, the hearts from the obese HFD MRL mice metabolically adapt and thereby prevent DCM. The HFD MRL hearts completely down-regulate their carbohydrate metabolism pathways and shift to lipid metabolism. This adaptation strategy works well for the MRL mice as they do not develop DCM despite pronounced weight gain, especially in the usually pathogenic visceral fat deposits. It is these experimentally identified metabolic adaptations that will inform future therapies for patients suffering from DCM.

Initially we hypothesized that, like the skeletal muscles [9] and liver tissues (Gonzalez M., unpublished observations), the HFD MRL cardiac tissues would have increased levels of pAMPK. Surprisingly, our original hypothesis was not supported by the data. Instead the data identifies that the HFD MRL cardiac tissues have decreased pAMPK. Despite this being opposite numerous published manuscripts, this pAMPK reduction is correlated with improved metabolic flexibility and improved function in the obese HFD MRL mice.

## Methods

### Animal protocols

The experiments and procedures in this study were approved by the University of Illinois at Chicago (UIC) Institutional Animal Care and Use Committee (IACUC). Male mice from the C57BL/6J (B6), and MRL/MpJ (MRL), with an intact Fas gene, strains were housed in the same animal facility on a 14 h light, 10 h dark cycle with water ad libitum. At 3 weeks of age, mice were assigned either a chow (CD, 7012 Teklad LM-485 Mouse/Rat Sterilizable Diet, Harlan Laboratories, Indianapolis, IN, USA) or high fat diet (HFD, 60 % kcal from fat, D12492 Research Diets Inc, New Brunswick, NJ, USA). This ad libitum diet was maintained for 12 weeks through the end of the experiment. The mice were euthanized following UIC and nationally accepted protocols with prolonged carbon dioxide exposure followed by cervical dislocation. Tissue samples were harvested immediately. Samples designated for immunoblotting were flash frozen in liquid nitrogen and stored at −80 °C until processed. Tissue samples for immunofluorescence were placed in optimal cutting temperature (OCT) compound and then frozen in liquid nitrogen cooled isopentane.

### Echocardiography

Echocardiography was performed by Dr. Robert Gaffin of the UIC Center for Cardiovascular Research. Mice were anesthetized using 3 % isoflurane, which was decreased to 2 % for anesthesia maintenance. Cardiac morphological, diastolic, and systolic parameters were assessed using the VisualSonics Vevo 770 (VisualSonics, Toronto, Canada) with a RMV707B (30 MHz) scanhead and the built-in echocardiography software.

### Histology and immunofluorescence

The previously frozen tissues were sectioned at seven microns on a cryostat. Tissue samples for Picro Sirius Red were sectioned at 20 microns and processed by the UIC Research Resources Center (RRC) Histology Core. Heart sections from all four groups were placed on the same slide to minimize artifacts. ImageJ was utilized to quantify the percentage of red staining per image.

The slides for immunofluorescent staining were fixed for 20 min in −20 °C MetOH, washed 3 times in 4 °C PBS for 3 min, and blocked in 5 % FBS in PBS for 15 min at room temperature. The slides where incubated with anti-γ sarcoglycan (1:100, Novocastra Labs, Buffalo Grove, IL, USA), anti-Glut4 (1:50, EMD Millipore, Darmstadt, Germany) or anti-fibronectin (1:100, Sigma, St. Louis, MO, USA) in PBS with 5 % fetal bovine serum for 1 h at room temperature, then aspirated and washed three times for 15 min in 4 °C PBS. The secondary antibodies (Invitrogen) at 1:500 in 5 % FBS in PBS were placed onto the slide for 1 h at room temperature in the dark. After another series of washes, the slides were mounted using Vectashield mounting solution, with DAPI (Vector Laboratories, Burlingame, CA, USA). Heart sections from all four slides were affixed to a single slide to minimize artifacts. ImageJ was used to get the average intensity of staining across each section or along a line segment.

### Cardiomyocyte cell size quantification

Cardiomyocyte cell size was quantified by highlighting the γ-sarcoglycan staining on the tissue images using a combination of GIMP (http://www.gimp.org/) and ImageJ (http://rsbweb.nih.gov/ij/). In brief, the stain was outlined and used to generate an image of uniformly black cells on a white background. The minimum diameter was then quantified in ImageJ and used to calculate area. One septal image (100× initial magnification) per animal, and at least four animals per group were quantified; the averages per group were then statistically compared.

### Immunoblot analysis

Expression levels of specific proteins were assessed by immunoblotting. Each tissue sample was added to 250 μl lysis solution (20 mM HEPES pH 7.4, 10 mM NaF, 50 mM β-glycerol phosphate, 2 mM EGTA, 1 % Triton X100, 10 % glycerol, 2.5 μl of sodium orthovanadate, 2.5 μl of DTT, 2.5 μl of Halt Protease and Phosphatase Inhibitor Cocktail (100×, Thermo Scientific, Hanover Park, IL, USA). The mixture was homogenized on ice using a TissueRuptor (Qiagen, Venlo, Netherlands). The solution was then spun at 4 °C for 10 min. Supernatant protein concentrations were determined by Bradford assay (Thermo Scientific Coomassie, Bradford Protein Assay Kit). Fifty ug of protein were heated for 5 min at 100 °C and loaded onto 12 % acrylamide gels (Mini-Protean TGX, Biorad, Hercules, CA, USA). Gels were transferred onto PVDF membrane using a semi-dry transfer system (iBlot, Invitrogen, Camarillo, CA, USA). Five percent reconstituted non-fat dairy milk (NFDM) in tris-buffered saline with 0.1 % tween-20 (TBST) was used to block the gels for 15 min. The membranes were incubated at 4 °C overnight in 5 % NFDM/TBST with primary antibodies [anti-AMPK, anti-pAMPK, and PGC-1α (1:1000, Cell Signaling Technology, Danvers, MA, USA); anti-ACC, anti-pACC, fibronectin (1:500, Santa Cruz Biotechnology, Santa Cruz, CA, USA); anti-HKII (1:5000, Millipore, Billerica, MA, USA); VDAC, Oxidative Phosphorylation antibody cocktail (1:1000, Abcam, Cambridge, MA, USA), Glut4 (1:500, EMD Millipore, Darmstadt, Germany), and γ-sarcoglycan (1:1000, Novocastra Labs, Buffalo Grove, IL, USA)]. Three 15-min washes with 1× TBST were performed after the primary antibody was removed. Blots were then incubated at room temperature for 1 h with horseradish peroxidase (HRP) conjugated species specific secondary antibodies (Invitrogen, 1:2500 in 1× TBST). Following another set of washes bound HRP was detected with ECL Prime (Amersham, GE Healthcare Bio-Sciences AB, Uppsala, Sweden) and a Chemidoc (Biorad Life Sciences Group, Hercules, CA, USA). Protein bands at the appropriate size from at least six animals per group were quantified using ImageJ. Ponceau staining was used to verify loading for each gel and γ-sarcoglycan, a membrane localized protein, immunoblotting of sister gels was used for normalization. To compare across gels, bands from each gel were normalized to the CD B6 average for that protein.

### Electron microscopy

Freshly isolated heart apexes from one animal of each group were delivered to the electron microscopy facility of the UIC RRC. Micrographs were captured in a blinded manner at 11,600× and 34,400× magnifications.

### Quantitative PCR

Previously published methods were used to quantify the amount of mitochondrial genome represented by subunit II of cytochrome C and using the nuclear gene succinate dehydrogenase subunit A as a control [13, 18].

### Statistics

Student t tests were performed in Microsoft Excel comparing the appropriate groups. Values <0.05 were considered significant between the groups being examined.

## Results

We subjected the MRL and control B6 mouse strains to 12 weeks of high fat diet (HFD) or control diet (CD) to investigate the abilities of the MRL mouse hearts to metabolically adapt to HFD. The HFD fed MRL mice gained significantly more weight than the CD fed MRL mice [9], and the HFD MRL mouse hearts demonstrated altered, beneficially adapted metabolic changes. Because the HFD mouse model mirrors human T2DM, identifying and investigating these beneficial metabolic changes will be essential for future diabetic cardiomyopathy (DCM) therapies.

### HFD MRL mouse hearts are protected against high fat diet-induced cardiac remodeling

**Fig. 1:**
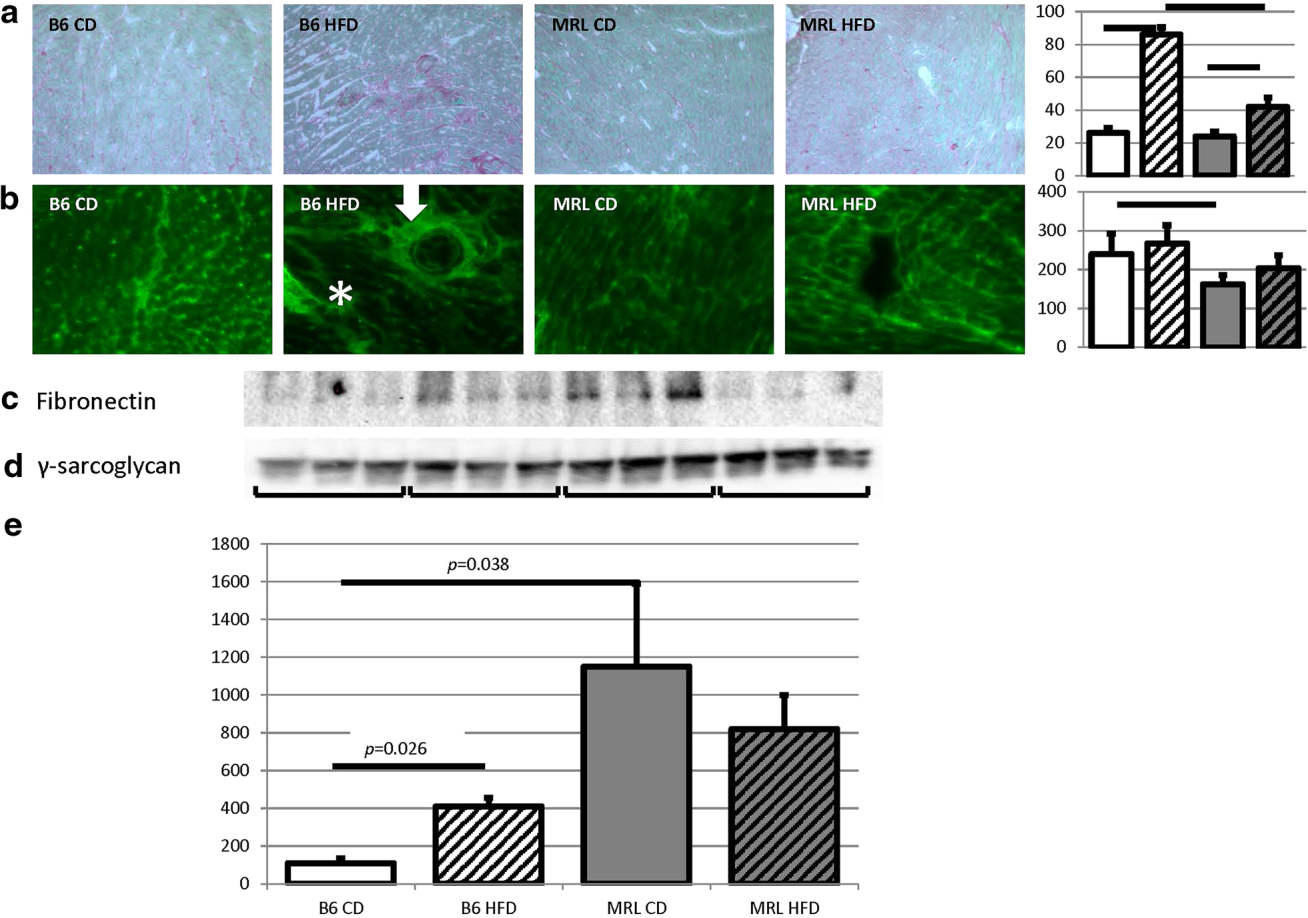
The HFD B6 hearts present with fibrosis, which is absent in the HFD MRL hearts. **a** Picro Sirius Red staining was enhanced in the HFD B6 hearts compared to the other three groups. Initial magnification ×100. Histogram of ImageJ quantification follows, N = 3, *bar* represents *p* < 0.05. **b** Representative immunofluorescent microscopy of fibronectin stained tissues confirms the increased fibrosis in HFD B6 hearts. Initial magnification ×100. Histogram of ImageJ quantification follows, N = 3, *bar* represents *p* < 0.05. **c** Immunoblot of fibronectin verifies increased fibrosis in the HFD B6. **d** Control γ-sarcoglycan immunoblot. **e** Quantification (N = 6, B6 CD versus B6 HFD *p* = 0.026, B6 CD versus MRL CD *p* = 0.038)

The hearts from the four mouse groups (CD B6, HFD B6, CD MRL and HFD MRL) were analyzed with histologic and immunofluorescence staining. The HFD B6 hearts displayed increased red staining with Picro Sirius Red, a fibrosis indicator, when compared to the three other groups (Fig. 1a). The Picro Sirius Red images were quantified and demonstrated a significant increase of collagen in the HFD B6 cardiac tissue (Fig. 1b). Similarly, when stained with fibronectin, the HFD MRL hearts stained comparably to the B6 CD and MRL CD hearts; while the HFD B6 hearts contained many regions of positive staining, further demonstrating increased fibrosis (Fig. 1c). In particular, the HFD B6 hearts contained numerous instances of perivascular fibrosis, an early stage in fibrosis progression (arrow in Fig. 1c) and interstitial fibrosis, a more advanced stage of the fibrotic process (asterisk in Fig. 1c). These images were also quantified and indicated an increase of fibronectin in the B6 HFD hearts (Fig. 1d). To quantify the increased fibrosis identified in the HFD B6 mice, an immunoblot of fibronectin was also performed. In support of the histology and immunofluorescence, the immunoblot identified that the HFD B6 mice had significantly increased fibrosis (B6 CD versus B6 HFD *p* = 0.02, immunoblot Fig. 1e, and quantified in 1f). Interestingly, this characteristic of cardiac remodeling was absent in the HFD MRL hearts when compared to their CD counterparts. Surprisingly, both groups of MRL hearts trended to an increase in fibronectin when compared to the B6 mice. Based upon these initial intriguing findings, we pursued cardiac characterization of the metabolically unique MRL mice fed the HFD. Identification of the mechanism causing HFD cardiac fibrosis resistance is of the utmost importance for obese and T2DM patients.

**Fig. 2:**
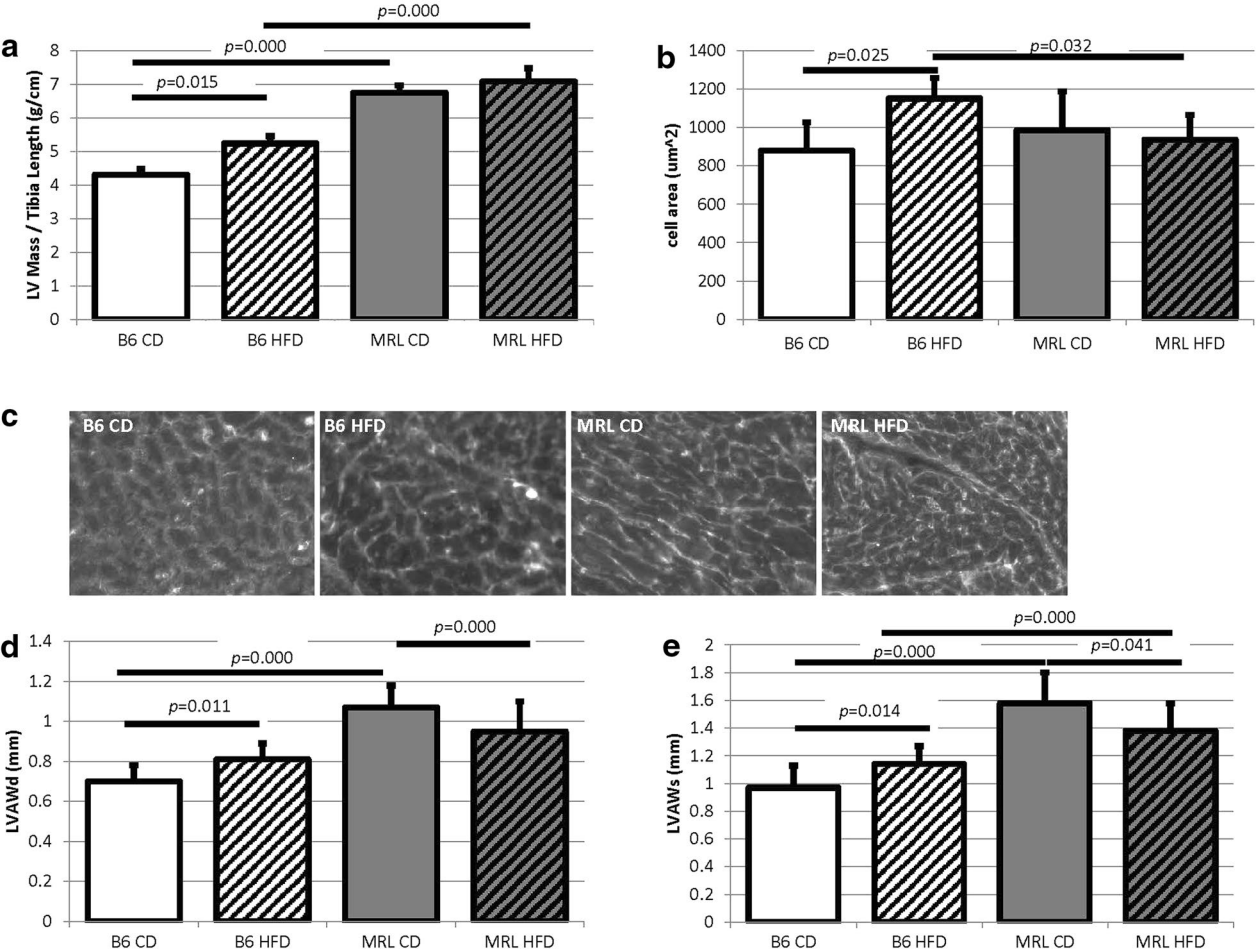
The hearts from HFD B6 mice display morphologic pathologies. **a** The echocardiography determined left ventricular (LV) mass normalized to tibia length is larger in the HFD B6 mice when compared to the CD B6 mice (*p* = 0.015, N > 8). **b** Similarly, the immunofluorescent determined cell size is larger in the HFD B6 mice when compared to the three other mouse groups (B6 CD versus B6 HFD, *p* = 0.025, B6 HFD versus MRL HFD *p* = 0.032, N > 3). **c** Representative cell sizes visualized by γ-sarcoglycan staining. **d**, **e** Echocardiography also revealed increases in left ventricle anterior wall during diastole and systole in the HFD B6 hearts compared to the CD B6 hearts (N > 8, *bars* indicate *p* < 0.05)

To further investigate cardiac remodeling, hypertrophy was evaluated by (1) left ventricle mass (obtained from echocardiography) over tibia length and (2) cardiomyocyte size (quantified on immunofluorescent staining). Only the HFD B6 animals demonstrated pathologic cardiac hypertrophy as their left ventricle masses/tibia lengths significantly increased over the B6 CD controls (Fig. 2a). The HFD MRL left ventricle masses were not significantly larger than the CD MRL left ventricle masses. The MRL hearts are not pathologically enlarged; the size is a physiologic compensation for their larger body size. Tibia lengths were utilized as the normalizing factor instead of body weight because the HFD mice gained so much weight this normalization actually showed a decrease of cardiac size in the HFD mice. We also identified significantly larger cardiomyocytes in the HFD B6 mice compared to the three other groups (quantified in Fig. 2b and representative images Fig. 2c). The B6 HFD cardiomyocytes were larger than their CD counterparts (1152 ± 108 versus 880 ± 148 μm^2^, *p* = 0.025, N > 3) and larger than the HFD MRL cells (1152 ± 108 versus 937 ± 130 μm^2^, *p* = 0.032, N > 3).

Further evidence of cardiac remodeling in the B6 HFD mice comes from additional echocardiography morphometric characteristics. The left ventricular anterior wall during diastole (LVAWd) and during systole (LVAWs) were both significantly increased in the B6 HFD fed animals compared to the B6 CD mice (Fig. 2d, e; Table 1). This is indicative of the expected hypertrophic cardiac remodeling in response to HFD. The HFD MRL mice however showed significant decreases in these two morphologic characteristics compared to their CD MRL counterparts. Other morphometric values that were obtained through echocardiography were not significantly different between the diets within either strain (Table 1). Future investigations will identify the beneficial metabolic adaptations in the MRL heart tissue for future therapies in humans.

**Table 1:** Echocardiography parameters for the four mouse groups

	B6 CD	B6 HFD	MRL CD	MRL HFD
Morphological				
LVAWd (mm)	0.70 ± 0.08	*0.81* *±* 0.08*	1.07 ± 0.11	*0.95* *±* 0.15*
LVAWs (mm)	0.97 ± 0.16	*1.14* *±* 0.13*	1.58 ± 0.22	*1.38* *±* 0.20*
LVIDd (mm)	3.87 ± 0.29	3.91 ± 0.28	4.12 ± 0.25	4.17 ± 0.34
Diastolic				
E DT (ms)	20.04 ± 3.69	19.63 ± 4.15	13.59 ± 5.02	19.04 ± 5.77
E/A	1.55 ± 0.32	1.81 ± 0.72	2.00 ± 0.55	2.13 ± 0.52
E′/A′	1.05 ± 0.31	1.18 ± 0.61	1.33 ± 0.27	*1.03* *±* 0.16*
EDV (μL)	65.86 ± 12.29	67.84 ± 11.24	75.54 ± 10.05	78.26 ± 13.37
Systolic				
EF %	56.33 ± 4.25	56.67 ± 8.84	70.25 ± 8.97	64.63 ± 11.30
FS %	29.03 ± 2.69	29.58 ± 6.67	39.91 ± 7.59	35.74 ± 8.94
HR	439.00 ± 40.69	448.77 ± 54.40	441.90 ± 54.45	441.08 ± 35.52

Italics indicate significance (*p* < 0.05) versus the control diet group of the same strain

N > 14

*LVAWd* left ventricular anterior wall in diastole, *LVAWs* in systole, *LVIDd* interior diameter in diastole, *E DT E* wave deceleration time, *EDV* left ventricular end diastolic volume

### MRL mouse diastolic function apparently benefits from high fat diet-induced obesity

The MRL left ventricles responded favorably to the HFD as the echocardiographic parameters indicate increased pliability and less fibrosis. E wave deceleration time (E DT) is an inverse measure of left ventricle stiffness; increasing deceleration time indicates decreasing stiffness. MRL HFD animals had significantly longer E DT times than their chow diet controls (*p* = 0.03, Table 1), while there was no difference between the B6 CD and B6 HFD animals. From this data it also appears that the MRL CD hearts are more rigid as their E DT was significantly lower than the B6 CD hearts (*p* = 0.0068, Table 1).

The E′/A′ ratio is an echocardiographic measure of left ventricular filling. There was no difference in the E′/A′ ratio between the two B6 groups. MRL mice on the HFD diet however had a significantly lower ratio than their CD counterparts (*p* = 0.004, Table 1). This decrease brought the E′/A′ ratio of the HFD MRL mice close to the chow B6 ratio, bringing the ratio to a more normative level. From the E′/A′ ratio it again appears as though the MRL hearts are benefited by the HFD.

Neither ejection fraction (EF) nor fractional shortening (FS) were affected in either mouse strain by the HFD (Table 1). Both of the MRL animal groups had higher EF and FS than the B6 animals but this is simply physiological, again due to the larger MRL animals. In addition, neither mouse strain developed hypertension as a result of the 12 weeks of HFD when compared to the age matched CD counterparts (data not shown). The lack of hypertension is surprising in light of the HFD-enhanced weight gain displayed by both mouse strains.

### MRL cardiac tissues have lower activated pAMPK levels

**Fig. 3:**
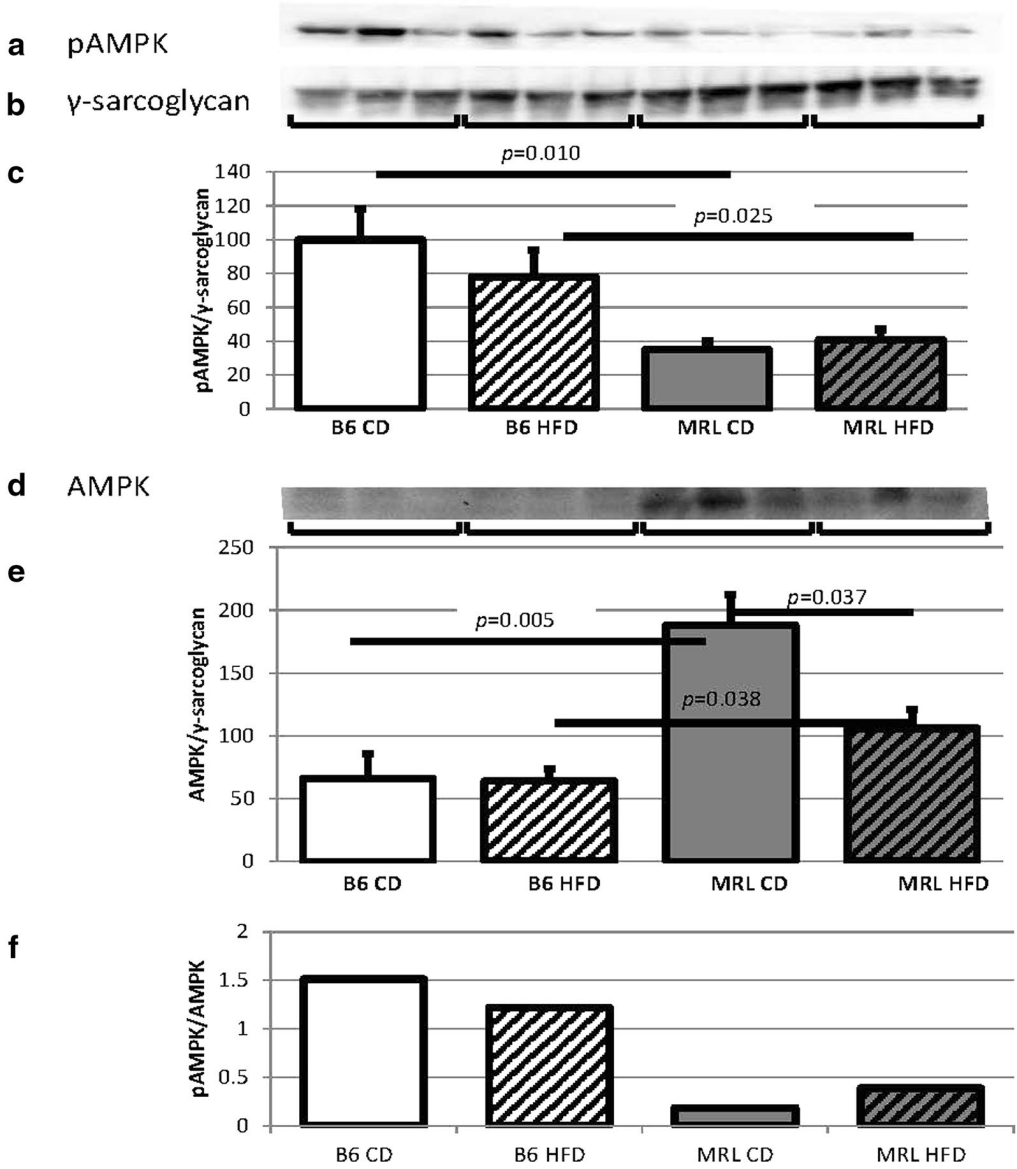
MRL hearts contain reduced amounts of pAMPK and AMPK. **a** Representative immunoblot of the phosphorylated α subunits of AMPK in mouse hearts. **b** γ-Sarcoglycan loading control. **c** pAMPK quantification normalized to γ-sarcoglycan and the B6 CD average (N = 15, B6 CD versus MRL CD p = 0.010, B6 HFD versus MRL HFD p = 0.025). **d** Representative immunoblot of the α subunits of AMPK in mouse hearts. **e** AMPK quantification normalized to the same γ-sarcoglycan blot as above and the CD B6 average (N = 9, B6 CD versus MRL CD *p* = 0.005, B6 HFD versus MRL HFD *p* = 0.038, MRL CD versus MRL HFD *p* = 0.037). **f** pAMPK/AMPK (N = 6)

AMPK and its activated form phosphorylate AMPK (pAMPK) function as sensors of available ATP levels in cells. AMPK is phosphorylated to pAMPK when the ratio of ADP to ATP shifts in favor of the lower energy ADP. pAMPK in turn facilitates energy production and energy conservation [19]. CD MRL mice have significantly lower levels of cardiac pAMPK than their CD B6 counterparts (*p* = 0.010, blot in Fig. 3a, control blot in Fig. 3b and quantified in Fig. 3c). This pattern of decreased pAMPK in MRL cardiac tissue remains at the conclusion of the HFD protocol: the HFD MRL cardiac tissues have significantly lower levels of pAMPK than the HFD B6 animals (*p* = 0.025). No significant difference was seen in either strain between the CD and HFD animals.

Although few examples of transcriptional regulation of AMPK exist, our current data proves that this does occur in cardiac tissues. The MRL hearts have significantly higher levels of AMPK alpha chains than the B6 hearts for both diet groups (Fig. 3d, quantified and normalized to γ-sarcoglycan in 3e, B6 CD versus MRL CD *p* = 0.005 and B6 HFD versus MRL HFD *p* = 0.038). In addition, HFD decreases the AMPK alpha chains in the MRL groups (*p* = 0.025). Identifying the transcription factors which modulate AMPK subunit expression will also prove useful in therapies directed against multiple human diseases. Although AMPK appears to be under transcriptional control in the cardiac tissue we also present the pAMPK to AMPK ratios. Both groups of B6 mice contained more pAMPK/AMPK than the MRL cardiac tissues (Fig. 3f).

### Protein levels indicate that MRL hearts perform decreased carbohydrate metabolism

**Fig. 4:**
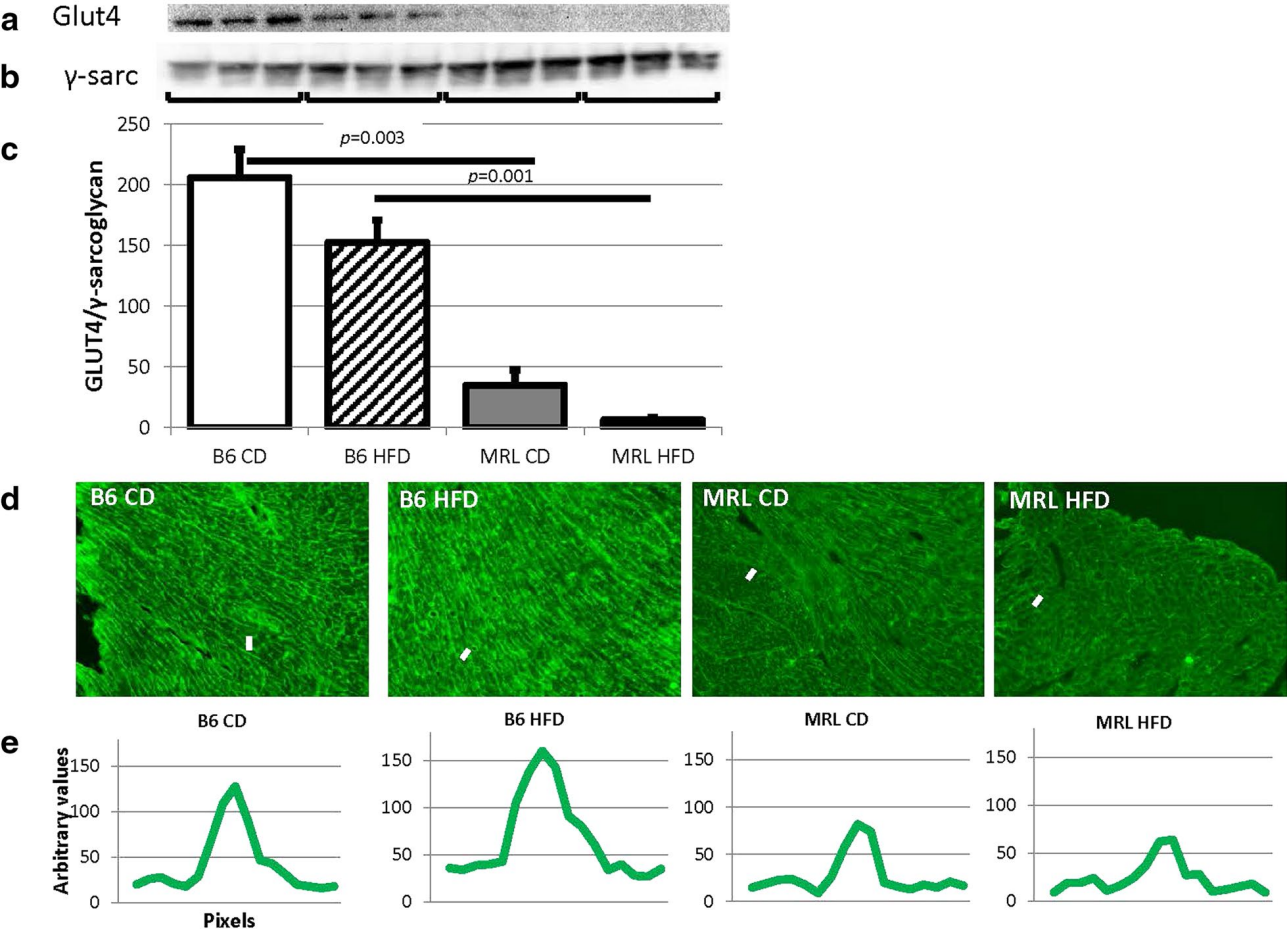
Glut4 is significantly reduced in the MRL hearts. **a** Representative immunoblot of Glut4 in mouse hearts. **b** γ-Sarcoglycan loading control. **c** Glut4 quantification normalized to γ-sarcoglycan (N = 9, B6 CD verses MRL CD *p* = 0.003, B6 HFD versus MRL HFD *p* = 0.001). **d** Representative immunofluorescence of Glut4 in the cardiac septum, original magnification ×100. **e** Brightness intensity of *lines* drawn (*white dashes* in Fig. 5d) across the plasma membranes

MRL CD mice were found to have significantly lower levels of total Glut4 protein present in their hearts than the B6 CD animals (*p* = 0.003, Fig. 4a). The HFD hearts from both strains contained less Glut4 than the CD hearts, with the HFD MRL animals containing the least (*p* = 0.001 versus the HFD B6 mice, Fig. 4a, control γ-sarcoglycan in Fig. 4b, and quantified in Fig. 4c). Because membrane localization of Glut4 is critical for its function heart tissue immunofluorescence was undertaken on the four mouse groups. The B6 heart cells demonstrated increased Glut4 membrane localization compared to the MRL groups (representative images in Fig. 4d, quantified in Fig. 4e). We quantified the signal intensity along a line crossing the plasma membrane (white lines in the Fig. 4d images). We reasoned that this intensity would reflect membrane localization of Glut4. The line graphs show increased membrane signal in the B6 hearts (Fig. 4e) compared to the MRL hearts.

**Fig. 5:**
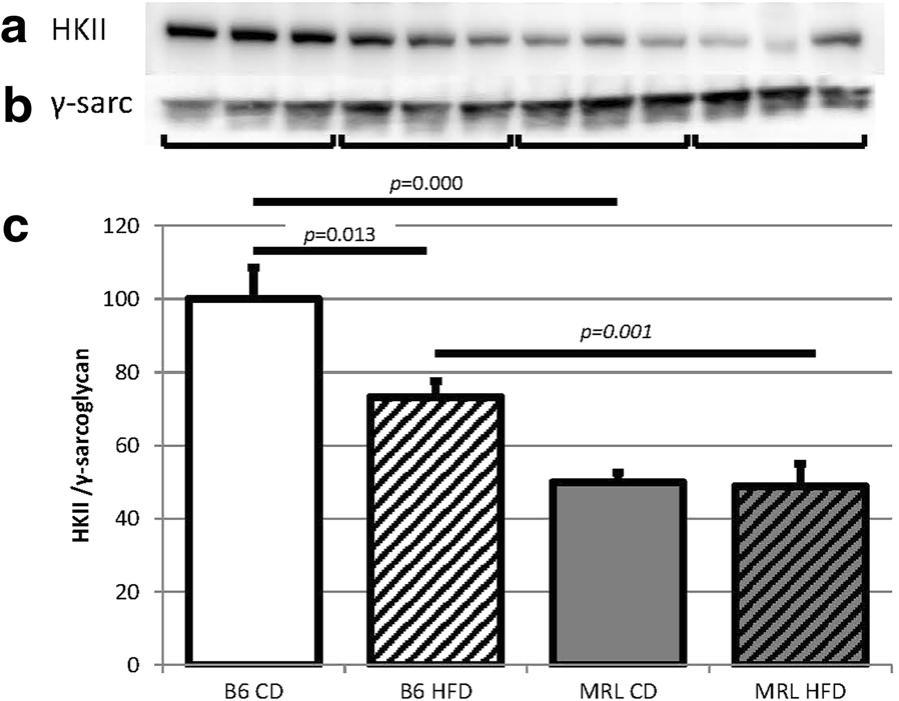
Hexokinase II is also reduced in the MRL cardiac tissue. **a** Representative immunoblot of HKII in the heart tissues. **b** γ-Sarcoglycan loading control. **c** Quantification of HKII normalized to γ-sarcoglycan and B6 CD quantities (N = 12, B6 CD verses MRL CD *p* = 0.000, B6 HFD versus MRL HFD *p* = 0.001, and B6 CD versus B6 HFD *p* = 0.013)

Supporting decreased carbohydrate metabolism, the CD MRL mice were also found to have lower hexokinase II (HKII) levels than the CD B6 mice (*p* = 0.000, blot Fig. 5a, γ-sarcoglycan control Fig. 5b and quantified in Fig. 5c). The HFD MRL mice also had significantly decreased HKII compared to the HFD B6 mice (*p* = 0.001). The B6 mice fed a HFD had lower levels of HKII than their chow counterparts (*p* = 0.013). The MRL mice demonstrated no difference in HKII levels between the CD and HFD cohorts.

### MRL hearts preferentially utilize fatty acid metabolism

**Fig. 6:**
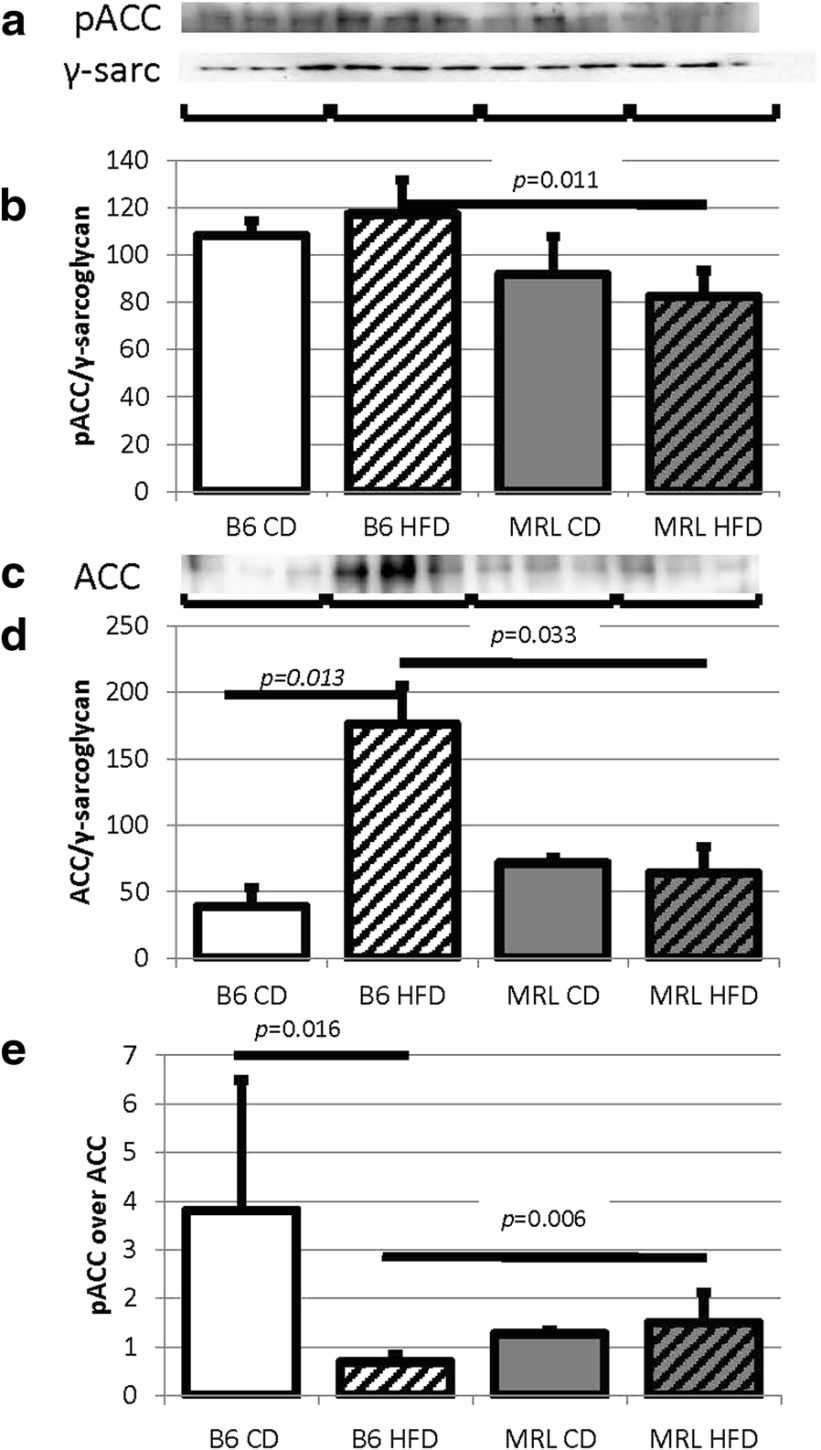
Decreased MRL cardiac ACC indicates increased fatty acid metabolism. **a** pACC immunoblot. **b** Quantification normalized to γ-sarcoglycan shows the MRL tissues have reduced pACC (N = 6, B6 HFD versus MRL HFD *p* = 0.011). **c** ACC immunoblot. **d** Quantification normalized to γ-sarcoglycan shows the MRL tissues have reduced ACC (N = 6, B6 HFD versus MRL HFD *p* = 0.033, B6 CD versus B6 HFD *p* = 0.013)

As the CD MRL hearts have less carbohydrate metabolic proteins, we expected an increase in the proteins of the fatty acid metabolic cascade. We therefore assessed the levels of acetyl-CoA carboxylase (ACC and pACC), the key regulatory protein in the fatty acid metabolic process. Decreased ACC decreases the amount of malonyl-CoA, ultimately increasing CPT1-mediated fatty acid transport into the mitochondria [2]. By immunoblot we found a significant decrease of ACC in the HFD MRL hearts compared to the HFD B6 hearts (immunoblot Fig. 6c, quantified in Fig. 6d, *p* = 0.033) indicating a likelihood of increased fatty acid oxidation in the MRL HFD hearts. Because of the large increase of ACC in the B6 HFD hearts compared to the B6 CD hearts (*p* = 0.013), we can also reason that the B6 HFD hearts are saturated with free fatty acids and have therefore decreased their fatty acid imports. These data are consistent with the MRL hearts performing less carbohydrate metabolism and increased β-oxidation in CD conditions and this propensity is increased in the HFD situation.

**Fig. 7:**
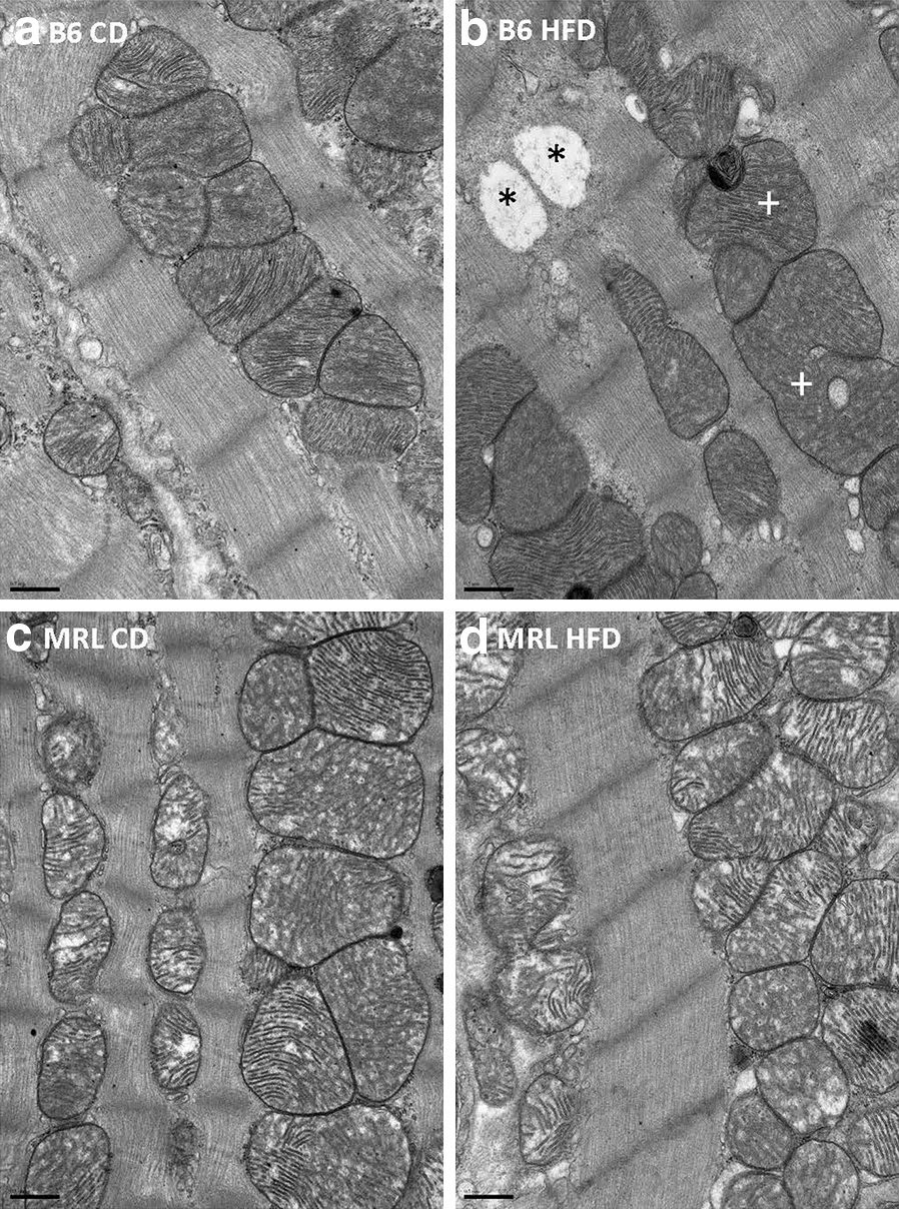
Representative electron micrographs of cardiac muscle from the septum identify lipid droplets in only the HFD B6 mice (*upper right panel*). The droplets were found cytoplasmically located (*asterisk*) or within the mitochondria (*plus*). *Bars* 0.5 μm

Hearts of HFD-fed animals usually store fat in the form of intracellular lipid droplets because they cannot metabolize all of the imported fat. By electron microscopy, cytoplasmic and mitochondrial lipid droplets are obvious in heart apex samples in the HFD B6 heart tissues, while the HFD MRL heart tissue is devoid of these lipid droplets (Fig. 7). The HFD MRL cardiomyocytes have successfully adapted to HFD metabolism without detectable pathologic cardiac consequences. In addition, the mitochondrial cristae of the HFD B6 hearts look malformed compared to the three other samples. All hearts were harvested and processed simultaneously, decreasing the chances of these differences being artifacts. The functional implications of the altered cristae are yet to be explored.

### MRL cardiac tissues have increased levels of mitochondrial DNA

**Fig. 8:**
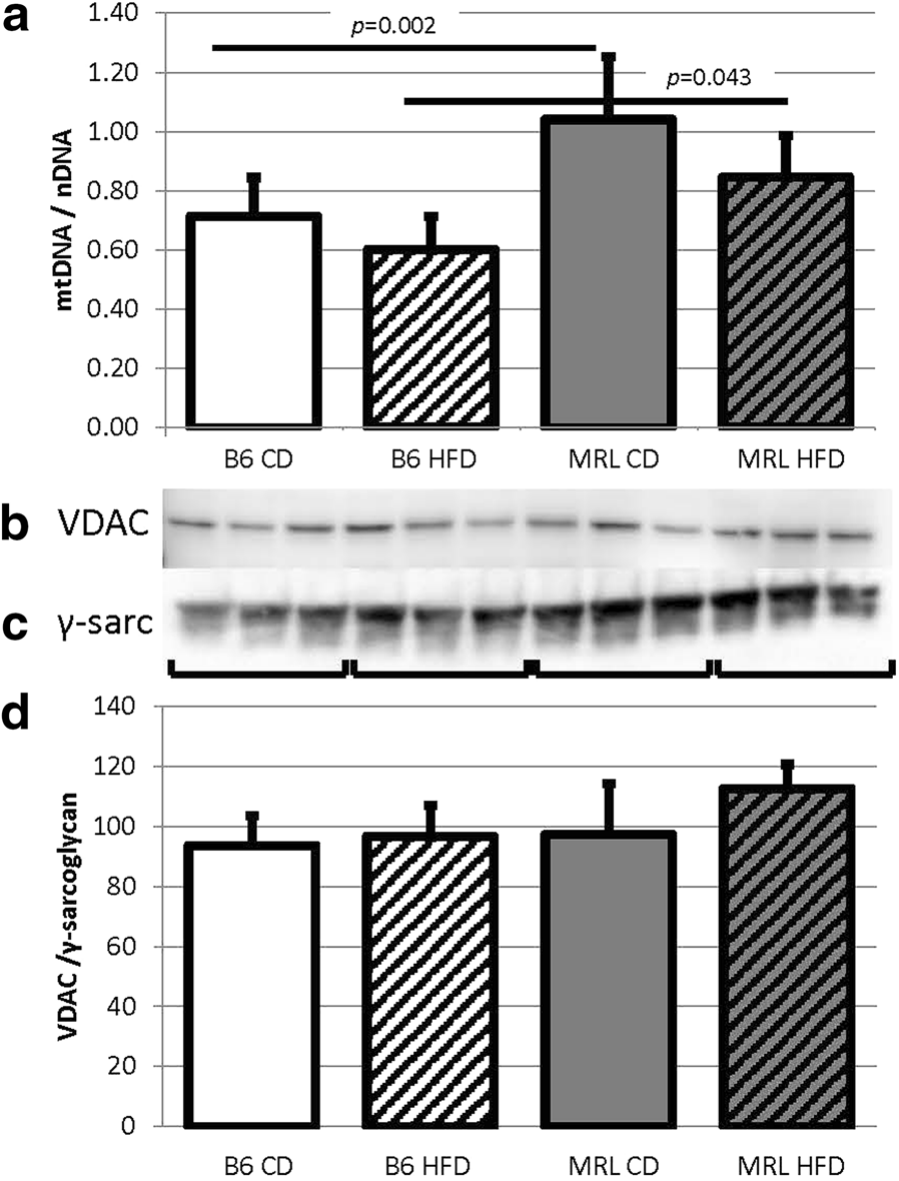
Mitochondrial differences in the two mouse strains. **a** The hearts from MRL mice contain increased mitochondrial genomes when normalized to nuclear DNA (N = 14, CD comparisons *p* = 0.002 and HFD comparisons *p* = 0.043). **b** However, quantification of VDAC immunoblots demonstrated no change in the outer mitochondrial membrane protein (N = 6). **c** Control to γ-sarcoglycan immunoblot. **d** VDAC normalized to γ-sarcoglycan (N = 6)

Quantitative PCR was used to compare the ratios of mitochondrial and nuclear genomes. The qPCR data indicated that the MRL hearts have increased mitochondrial genomes per nuclear genome when compared to the diet matched controls (Fig. 8a, CD B6 v CD MRL *p* = 0.002, HFD B6 v HFD MRL *p* = 0.043). A further method of quantifying mitochondria is to measure mitochondrial protein concentrations. Immunoblots of VDAC, a mitochondrial outer membrane protein were used. No differences were detected between the mouse groups in VDAC quantity (VDAC immunoblot Fig. 8b, γ-sarcoglycan control Fig. 8c, and quantified in Fig. 8d). To further analyze the mitogenesis portion of mitochondrial levels, PGC-1α levels were assessed in the hearts from the four mouse groups. No differences were detected between any of the groups (data not shown). The increase of mitochondrial DNA in the MRL hearts may be the proximal cause of the HFD resistant phenotype, this hypothesis remains to be determined by further experimentation.

### Electron transport chain (ETC) complexes are differentially affected by the HFD

**Fig. 9:**
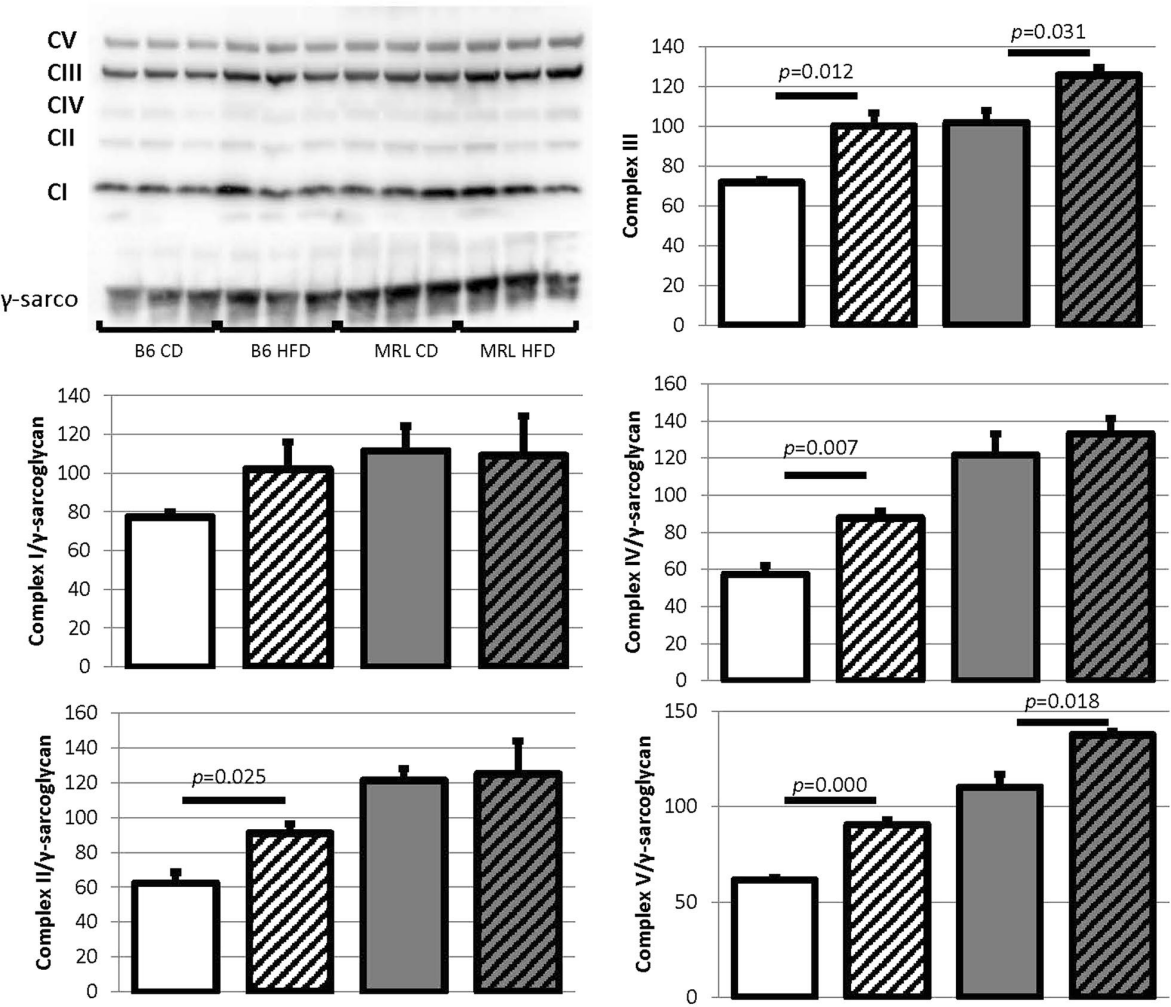
The B6 and MRL mouse strains respond differently to HFD mediated metabolic stress. Protein levels of single components of the electron transport chain’s complex I through V were assessed. The B6 hearts increased levels of all complex members, while the MRL hearts only significantly raised the levels of complex members III and V (N = 3)

As our hypothesis is that the MRL cardiomyocytes are chronically stressed energetically we also assessed one component of each ETC complex by immunoblot. In the control B6 mouse strain all assessed members (complex I subunit NDUFB8, complex II the 30 kDa subunit, complex III the core protein 2, complex IV subunit I, and complex V the alpha subunit) of the five ETC complexes were upregulated by HFD, and four of the five upregulated components are significantly increased (Fig. 9). The MRL hearts adapt to the HFD differently and only upregulated the complex III and complex V components significantly. We have not yet conducted activity assays, analyzed reactive oxygen content or quantified ATP levels in these hearts, so in-depth metabolic conclusions cannot yet be made.

## Discussion

The cardiac responses to HFD in the B6 and MRL wild type mouse strains were analyzed for DCM phenotype. As expected, the HFD B6 mice developed key features of DCM: hypertrophy, morphometric changes, and lipid accumulation. In contrast, despite gaining a similar amount of weight as the HFD B6 mice [9], the wild type MRL mice were resistant to the HFD and did not develop any features of DCM. Remarkably, the HFD MRL hearts contained significantly less pAMPK by immunoblot than the HFD B6 hearts. The MRL mice also reduced their carbohydrate metabolic enzymes while increasing the enzymes required for β-oxidation.

Multiple assays verified that the HFD B6 mice were susceptible to the diabetic cardiomyopathy (DCM) pathologies associated with HFD-induced obesity. The cardiac tissues were stained for various markers of fibrosis. By Picro Sirius Red staining the B6 HFD hearts contained the most positive red regions followed by the MRL HFD mice and then the CD control hearts contained the least. A very similar trend was seen when sections were stained for fibronectin. However immunoblots with the same fibronectin antibody provided inconsistent results. The ultimate reason for this discrepancy could be as simple as the denaturing conditions of the gel versus the largely unmodified proteins in the immunofluorescence. The discrepancy could also arise from immunofluorescence image selection. We feel this is unlikely as we chose the images using the blue (DAPI, nuclear) filter set and looking for blood vessels to compare perivascular fibrosis. The increased fibronectin by immunoblot in the CD MRL hearts may reflect the slight level of metabolic stress these hearts are subjected to, due to the mitochondrial heteroplasmies. Additional experiments are being initiated to identify the basal metabolic differences between these two mouse strains. Despite the inconsistent results, when considering all of the data it is apparent that the HFD B6 hearts are pathogenically affected, while the HFD MRL hearts are beneficially adapting to the HFD stress.

By echocardiography, the ratios of left ventricle mass to tibia length were increased in the HFD B6 compared to CD B6 mice. Tibia length was utilized as the normalizing factor because the HFD animals gained such a large amount of weight that this would misrepresent the heart mass increases. Tibia length did not change between the diets after the 12 weeks of diet intervention (data not shown). In addition, quantitation of histology sections demonstrated larger cardiomyocytes in the HFD B6 mice compared to their CD counterparts. Furthermore, additional morphologic differences revealed by echocardiography demonstrated increased values only in the HFD B6 mice compared to the CD B6 mice. In contrast, the HFD MRL mice were not statistically different than their CD counterparts in any of these parameters indicating the MRL resistance to HFD. By left ventricular size to tibia length ratios, hearts from both MRL diet groups were larger than the B6 hearts. This is a physiologic increase due to the larger MRL animal size [9] and not due to pathology because the size is not further increased by HFD.

The echocardiography data reveals a functional preservation and in some cases an improvement in the HFD MRL hearts. Although surprising, this protection may be caused by a chronic reduced mitochondrial efficiency [13, 18], due to two mitochondrial heteroplasmies identified in these mice [20]. Heteroplasmy describes the multiple copies of a mitochondrial genome which contains both wild type and polymorphic sequences. Therefore, this leads to chronic cardiomyocyte metabolic stress which in this situation is beneficially adaptable to the HFD. One of the manifestations of this stress is an increased glycolysis rate in the MRL cells compared to B6 heart cells when glucose is the substrate [18]. This data corroborates our results, which indicate that MRL cardiomyocytes favor lipids as substrates and actually functionally improve on a HFD. These data and this model are currently being further investigated.

Previous data indicate that most mice receiving a HFD will increase their lipid metabolism and decrease their carbohydrate metabolism [21]. The HFD fed MRL mice appear to take these metabolic shifts to an extreme. It has been proposed that pharmacologically reversing these changes; increasing glucose utilization and decreasing lipid metabolism will be beneficial to heart failure patients [21]. The health of the MRL mouse hearts indicates the opposite may be true and increased reliance upon lipid metabolism is the healthy adaptation. If these extreme metabolic shifts cause the HFD MRL hearts to remain healthy will be analyzed in future experiments.

The decreased level of AMPK and pAMPK in the healthy HFD MRL hearts is unexpected. A number of studies have demonstrated that increased pAMPK is usually associated with, or directly causes improved cardiac function [22]. There is also evidence that increased pAMPK benefits mouse models of DCM [23]. Importantly, Steinbusch et al. [24], showed that viral over-expression of a constitutively active AMPKα2 in primary cultures of insulin resistant cardiomyocytes significantly protected cellular metabolism. These authors also identified that increasing pAMPK activity does not correct all high lipid media consequences. For example, the cardiomyocytes with over expression of pAMPK maintained high palmitate uptake and triglyceride accumulation. The authors concluded that increasing pAMPK rectified glucose metabolism but not lipid metabolism. Our current data is consistent with other mechanisms beyond pAMPK activation controlling cardiac lipid metabolism. Knowledge of these mechanisms will provide additional therapeutic targets for humans suffering from T2DM and DCM.

**Fig. 10:**
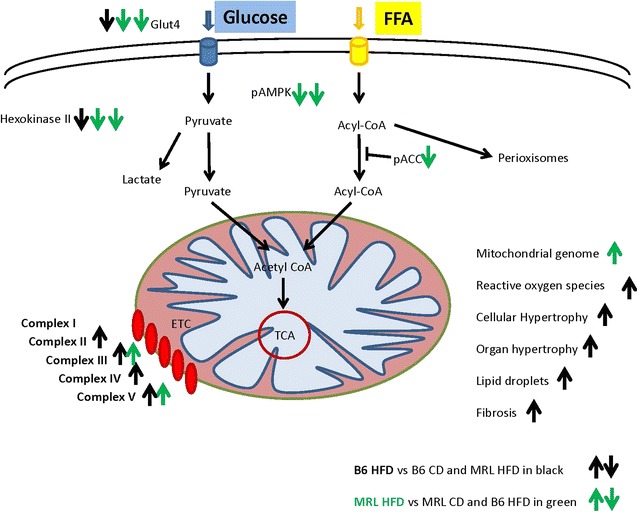
The working model of the differential response to HFD by the B6 and MRL mouse strains. *Black arrows* represent protein level changes in the B6 hearts and *green arrows* indicate protein level changes in the MRL hearts

These novel MRL pathways would cause the MRL hearts to favor lipid metabolism over carbohydrate metabolism to a larger extent than other mouse strains. By increasing their lipid metabolism, excess lipids would not be stored in the hearts causing lipotoxicity as usually occurs in HFD B6 fed mice [25]. We hypothesize that the CD MRL heart tissue is in a chronic state of metabolic stress due to less efficient electron transport chain activities as a result of the two mitochondrial heteroplasmies. Therefore we can view the MRL tissues as preconditioned for the HFD diets and completely adaptable to the added stress of a HFD. A schematic of this working model is presented in Fig. 10, black arrows indicating increase or decrease of indicated proteins for B6 HFD mice and green arrows indicating MRL HFD protein changes.

In support of the MRL hearts favoring lipid metabolism are the changes in key metabolic proteins between the two mouse strains. The MRL hearts, regardless of diet, have significantly reduced total and membrane localized Glut4. Glut4 is a key protein in cellular entry of glucose and its translocation to the cell membrane is a well-known function of pAMPK [26]. Therefore, the decreased Glut4 translocation is consistent with reduced pAMPK in the MRL hearts. A second key protein in carbohydrate metabolism is hexokinase II (HKII) which phosphorylates glucose and thereby locks it into the cell. HKII is also significantly reduced in the MRL hearts. Additional assessment of an important step in lipid metabolism revealed key proteins involved in inhibiting lipid transport to be downregulated in the MRL heart tissues. Combined, these data further support the hypothesis that the MRL hearts down regulate glucose metabolism and favor lipid metabolism more than B6 hearts. Therefore it is clear that the MRL hearts are metabolically adapting to the HFD stress and are responding to the pathogenic signals in favorable ways.

Previous reports indicate that MRL tissues contain increased mitochondrial genomes compared to B6 control strain [13, 18]. We sought to identify if the HFD caused further changes in mitochondrial genome quantity in the cardiac tissues. By qPCR we identified significantly increased mitochondrial to nuclear genome ratios in both MRL mouse groups. This data was not supported by VDAC—an outer mitochondrial membrane protein—expression. A possible explanation is that the MRL mitochondrial genomes are more densely arranged within the matrix, therefore the DNA content will increase without increasing the membrane protein amount. This could be a further indication of chronic low-level stress in the MRL cardiac cells.

There is much discussion whether murine HFD can cause DCM. Many publications demonstrate DCM after murine HFD feeding [8]. Mice on a chronic HFD gain more weight than their CD counterparts, develop insulin resistance, have aberrant responses to glucose tolerance tests and insulin tolerance tests. Most mouse strains also respond to HFD with cardiac remodeling, which can be diagnosed with increased heart mass, cardiomyocyte hypertrophy, and cardiac fibrosis [8]. Other publications indicate no murine cardiac dysfunction after lengthy HFD, although in this example the HFD hearts did respond with morphologic changes [27]. Furthermore, it is becoming obvious that the type of fat in a HFD will differentially affect cardiac morphology and function during periods of stress, increasing specific fat intake often proving beneficial [28]. In the current manuscript, the 12 weeks HFD B6 control mice display certain features of DCM, such as hypertrophy, functional changes and lipid accumulation. In contrast, the HFD MRL mouse hearts successfully metabolically adapted to the HFD and did not display any characteristics of DCM.

## Conclusions

As diabetic cardiomyopathy (DCM) causes the majority of diabetic mortality, identification of a novel mouse model which does not succumb to DCM is an important scientific finding. In contrast to the B6 mouse strain used as controls, 12 weeks of HFD did not cause cardiac fibrosis, hypertrophy or functional detriments in the MRL hearts. The MRL cardiac tissue successfully metabolically adapted to the 12 weeks HFD protocol.

There are many experiments currently underway to further identify the beneficial adaptation in MRL metabolism. The data gathered from all of these experiments will help identify therapeutic targets for patients suffering from DCM. These therapeutic targets are potentially in metabolic pathways, such as specific pAMPK and AMPK modulations in skeletal muscle and cardiac tissues.

Currently a longer—20 week—HFD protocol is being conducted. This will verify the MRL mouse strain’s resistance to chronic HFD. These mice will also be subjected to acute insulin challenge to investigate tissue specific insulin sensitivity and resistance and further the identification of tissue specific metabolic adaptations. Furthermore the tissues harvested from these mice will be analyzed for enzymatic activities of key metabolic enzymes, such as adipose triglyceride lipase, hormone sensitive lipase and citrate synthase.

Currently, we are also investigating the HFD-associated inflammation. The MRL mice are known to have disparate immune reactions in response to trauma [29, 30], even these MRL/MpJ mice that have an intact Fas signaling pathway are susceptible to autoimmune phenotypes as they age [31, 32]. Because inflammation is a well-known component of HFD-induced metabolic pathologies this is a very important direction for future investigations.

## Abbreviations

AMPK: AMP activated protein kinase
B6: C57Bl/6 mouse strain
CD: control diet
DCM: diabetic cardiomyopathy
HFD: high fat diet
MRL: MRL/MpJ mouse strain
pAMPK: phosphorylated AMPK
T2DM: type 2 diabetes mellitus
